# Insanity

**Published:** 1868-09

**Authors:** 


### INSANITY.

Very many persons are actually insane, without its ever being known, until after their death, when the aggregate of their conduct, enables the survivors to make up a truthful verdict. An over dwelling on any one idea is an insanity; an over devotion to any pleasure or gratification, or appetite, or indulgence, or pursuit, is an insanity. Many persons are as "crazy as a bed bug," if any one can measure the amount, on the subject of their ailments; dyspepstics particularly; they can write by the acre, and talk by the mile on that prolific theme; and on another a thousand times more so, that remarkable baby for example, although every body else can see at a glance that it it is the ugliest little wretch in existance. There is one kind of madness always perfectly beautiful in our eyes, Husband and wife madness, where generous and kindly words are almost sure to be spoken in any five minutes conversation with any near friend; not continued, but a a loving word or two. Reader! what subject are you mad about? think, and correct the insanity, before, like a dreadful whirlpool, it sweeps your reason away forever.

"Madam, I can't have you here; I am sick, and my wife is sick; I have no hay or corn for your horses; I have no servants; and I had rather be chained to a galley-oar than wait on you myself."

Thus wrote a man to his brother's widow, who once proposed to make him a visit. He lived on milk and vegetables and wore a coat of tanned calf skin. When he died, he left money enough to Harvard University, to found the professorship of Theory and Practice of Medicine. He is a madman who disinherits his wife if she marries again, and madder than all these are they who live here, as if there were no hereafter.

